# Supplementary material for: Identification of photoperiod-induced specific miRNAs in the adrenal glands of Sunite sheep (Ovis aries)
Source: Front Vet Sci. 2022 Jul 22;9:888207. doi: 10.3389/fvets.2022.888207 (PMC9354845; doi:10.3389/fvets.2022.888207)
Supplement: Supplementary file 1 [file Data_Sheet_1.ZIP › Supplementary file/miRDeep2 file.zip!/miRDeep2 file/Novel_Structure/SP42A1/Novel_276.pdf]

Provisional ID	:	NC_019470.2_16109
Score total	:	1.9
Score for star read(s)	:	-1.3
Score for read counts	:	0
Score for mfe	:	1.6
Score for randfold	:	1.6
Score for cons. seed	:	
Total read count	:	78
Mature read count	:	78
Loop read count	:	0
Star read count	:	0

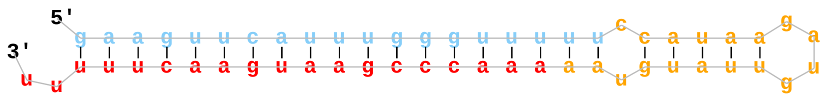

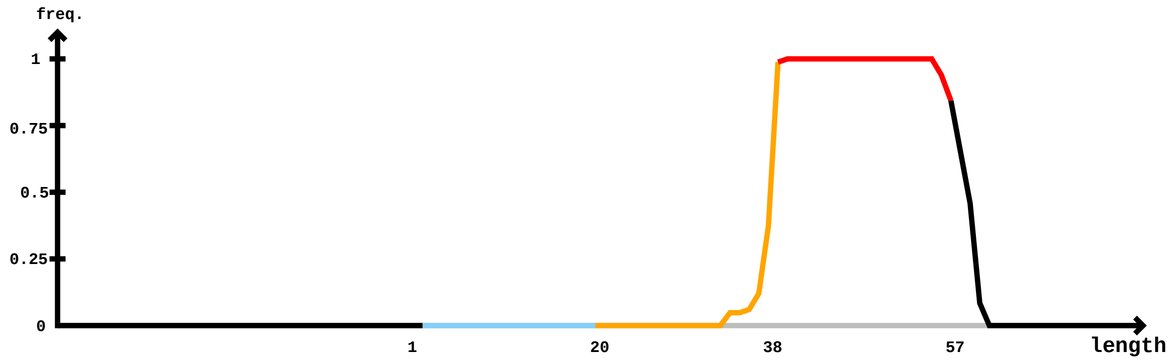

Star Mature

```
5'- uaauaauagauggccuuauaguaucagguuggcuaagaaguucauuuggguuuuuccauaagauguuauguaaaaacccgaaugaacuuuuugaucaguccaauagaaaag -3'   exp
    ........((((.........)))).((((((.((((((((((((((((((((((.(((((....))))).)))))))))))))))))))))).)))).))..........  reads   mm   sample
    ....................................................................ugGaaaaacccgaaugaacuuu.....................  4   1   seq
    ......................................................................uaaaaacccgaaugaacuuuu....................  1   0   seq
    .......................................................................aaaaacccgaaugaacuuu.....................  1   0   seq
    .......................................................................aaaaacccAaaugaacuuuu....................  2   1   seq
    .......................................................................aaaaaccAgaaugaacuuuuu...................  2   1   seq
    ........................................................................aaaaccAgaaugaacuuuu....................  2   1   seq
    ........................................................................aaaaccAgaaugaacuuuuu...................  4   1   seq
    ........................................................................aaaaccAgaaugaacuuuuug..................  2   1   seq
    ........................................................................aaaaccUgaaugaacuuuuuga.................  13  1   seq
    .........................................................................aaacccgaaugaacuuuu....................  3   0   seq
    .........................................................................aaacccgaaugaacuuuuu...................  19  0   seq
    .........................................................................aaacUcgaaugaacuuuuu...................  1   1   seq
    .........................................................................aaacccgaaugaacuuuuC...................  2   1   seq
    .........................................................................aaacccgaaugaacuuuuuC..................  1   1   seq
    .........................................................................aaaccUgaaugaacuuuuuga.................  1   1   seq
    .........................................................................aaacccgaaugaacuuuuugG.................  17  1   seq
    .........................................................................aaacccgaaugaacuuuuugGu................  7   1   seq
    ..........................................................................aCcccgaaugaacuuuuug..................  1   1   seq
```
